# *Ezh2* Regulates Early Astrocyte Morphogenesis and Influences the Coverage of Astrocytic Endfeet on the Vasculature

**DOI:** 10.1111/cpr.70015

**Published:** 2025-02-28

**Authors:** Xinghua Zhao, Mengtian Zhang, WenZheng Zou, Chenxiao Li, Shukui Zhang, Yuqing Lv, Libo Su, Fen Ji, Jianwei Jiao, Yufei Gao

**Affiliations:** ^1^ Department of Neurosurgery China‐Japan Union Hospital of Jilin University Changchun China; ^2^ Key Laboratory of Organ Regeneration and Reconstruction State Key Laboratory of Stem Cell and Reproductive Biology, Institute of Zoology, Chinese Academy of Sciences Beijing China; ^3^ Jilin Province Neuro‐Oncology Engineering Laboratory Changchun China; ^4^ University of Chinese Academy of Sciences Beijing China; ^5^ Jilin Provincial Key Laboratory of Neuro‐Oncology Changchun China; ^6^ Beijing Institute for Stem Cell and Regenerative Medicine Institute for Stem Cell and Regeneration, Chinese Academy of Sciences Beijing China; ^7^ Affiliated Hospital of Guangdong Medical University & Zhanjiang Key Laboratory of Zebrafish Model for Development and Disease Guangdong Medical University Zhanjiang China; ^8^ School of Medical Technology Tianjin Medical University Tianjin China

**Keywords:** astrocyte, autism, BBB, EZH2, morphogenesis

## Abstract

Astrocytes are crucial for central nervous system (CNS) development and function, with their differentiation being stringently controlled by epigenetic mechanisms, such as histone modifications. Enhancer of Zeste Homologue 2 (EZH2), a histone methyltransferase, is essential for the suppression of gene expression. However, the role of EZH2 in astrocyte early morphogenesis has remained unclear. Using an astrocyte‐specific Ezh2 knockout (cKO) mouse model, we examined the effects of EZH2 deletion on astrocyte morphogenesis, blood–brain barrier (BBB) integrity and neurodevelopment. Loss of EZH2 led to increased glial fibrillary acidic protein (GFAP) expression, altered astrocyte morphology and reduced coverage of astrocytic endfeet on blood vessels, compromising BBB integrity. Vascular abnormalities, characterised by increased vascular density and smaller vessel diameter, mirrored compensatory changes seen in moyamoya disease. RNA‐sequencing and ChIP‐seq identified *Ddn* as a key upregulated gene in *Ezh2* ^*cKO*^ astrocytes, influencing cytoskeletal changes via the MAPK/ERK pathway. Behavioural analysis revealed autism‐like traits, such as reduced vocalisations, without significant anxiety‐like behaviour. These findings highlight EZH2 as a critical regulator of astrocyte function, with its disruption contributing to neurodevelopmental disorders. This study provides novel insights into the molecular pathways governing astrocyte differentiation and suggests EZH2 as a promising therapeutic target for gliomas and other CNS disorders.

## Graphical abstract

Ezh2‐deficient astrocytes showed morphologically more slender but sparse dendrites, resulting in reduced endfoot coverage and also causing disruption of the blood–brain barrier.

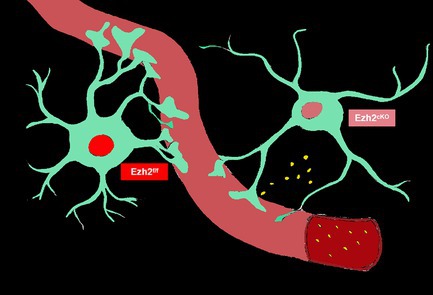

## 1 Introduction

Astrocytes, a key cell type in the central nervous system [1, 2, 3, 4, 5, 6, 7], undergo intricate developmental processes regulated by various molecular mechanisms, including epigenetic modifications [8, 9]. One of the hallmark genes in astrocyte differentiation is GFAP, whose expression is tightly regulated through epigenetic mechanisms such as DNA methylation and histone modifications [8, 10, 11]. These processes are essential for the proper maturation of astrocytes, ensuring their functional role in supporting neuronal activity and maintaining the BBB [2, 12, 13]. However, dysregulation of epigenetic mechanisms during astrocyte development has been linked to the onset of pathological conditions, such as gliomas [14, 15]. In particular, EZH2, a histone methyltransferase, is often overexpressed in gliomas, where it promotes tumorigenesis by silencing tumour suppressor genes and disrupting normal astrocyte differentiation [14, 16]. EZH2 is a key histone methyltransferase of the polycomb repressive complex 2, responsible for catalysing the trimethylation of lysine 27 of histone H3 (H3K27me3), thereby regulating gene transcription [17]. EZH2's role in regulating GFAP expression and astrocyte differentiation is of great interest, as it may offer insights into the molecular foundation of glioma formation and potential therapeutic strategies [18, 19, 20]. Understanding the interplay between epigenetic regulation and astrocyte development is crucial for elucidating the pathophysiology of gliomas and for developing novel strategies for targeting glioma progression.

Astrocytes also exhibit regional and cellular diversity within the CNS, contributing to the specialised functions of different brain areas [21]. Abnormalities in astrocyte morphology and function can lead to various neurodevelopmental and neurodegenerative diseases, such as obsessive‐compulsive disorder (OCD), autism spectrum disorders and multiple sclerosis [13, 22]. Recent studies have highlighted the role of epigenetic regulation in astrocyte differentiation and development, with the expression of key genes like GFAP being tightly controlled by mechanisms such as DNA methylation and histone modification [10, 15, 23]. These epigenetic changes are crucial for the proper maturation of astrocytes, ensuring their role in maintaining neuronal homeostasis and the integrity of the BBB [1, 2, 3, 6, 7]. Dysregulation of these epigenetic processes during astrocyte development has been linked to the onset of pathological conditions, including gliomas [24]. In particular, the histone methyltransferase EZH2 is often abnormally expressed in gliomas. Increased or reduced expression can disrupt normal astrocyte differentiation and promote tumorigenesis, and these tumour cells show obvious heterogeneity [19, 25]. Therefore, investigating the role of EZH2 in regulating GFAP expression and astrocyte differentiation may offer a deeper understanding of the molecular pathways involved in glioma development and reveal promising strategies for therapeutic intervention.

In this research, we used an *Ezh2* knockout mouse to explore the impact of EZH2 depletion in astrocytes. Our findings demonstrate that the absence of EZH2 in astrocytes results in significant morphological changes both in vitro and in vivo, with reduced astrocyte endfeet coverage of blood vessels, compromised BBB integrity and abnormal vascular development. Additionally, tight junction proteins were downregulated, further implicating EZH2 as a key regulator of astrocyte function and BBB maintenance. The significance of our study lies in its identification of EZH2 as a critical epigenetic regulator in astrocyte differentiation, providing new insights into how epigenetic modifications contribute to astrocyte function and the pathogenesis of neurological diseases. These findings may offer novel therapeutic avenues for targeting EZH2 in the treatment of glioma and other CNS disorders.

## 2 Results

### 2.1 *Ezh2* Deletion in Astrocytes Increases Cell Number and GFAP Expression

**FIGURE 1:**
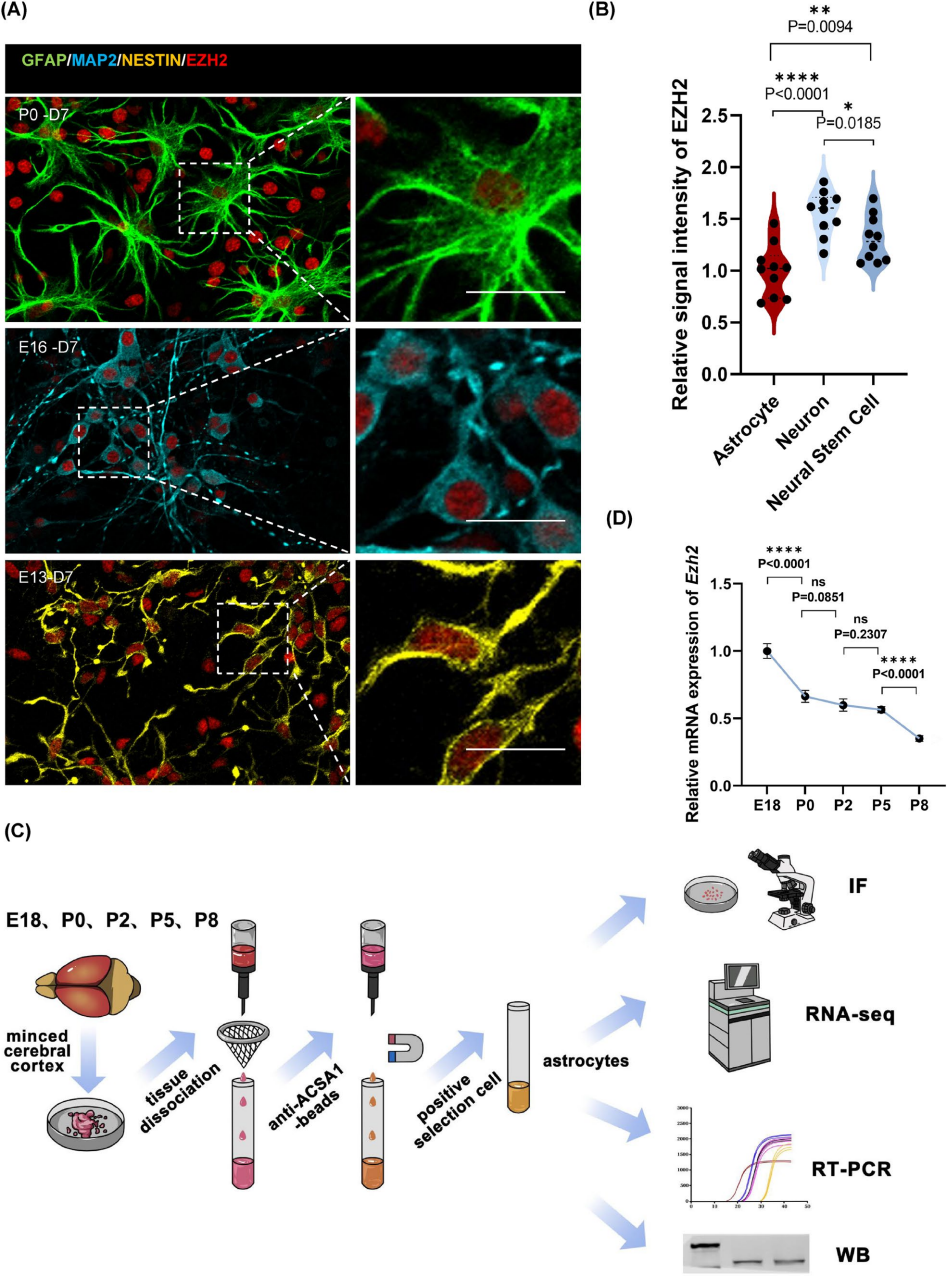
Ezh2 may play a critical role in the early morphological of astrocytes. (A) Immunofluorescent staining for EZH2 with astrocytes, neurons and neural stem cells of mice brains. Scale bar, 20 μm. (B) Statistic analysis of the relative fluorescence intensity of Ezh2 with astrocytes (P0, differentiation in vitro for 7 days), neurons (E16, differentiation in vitro for 7 days) and neural stem cells (E13, differentiation in vitro for 7 days) of mice brains, *n* = 10. (C) Schematic diagram of the purification of astrocytes from mouse cerebral cortex using magnetic bead screening technology. (D) Statistic analysis of the relative mRNA of Ezh2 in astrocytes of mouse cerebral cortex across different periods, *n* = 4.

Through a comprehensive analysis of the publicly available Tabula Muris database, we observed that *Ezh2* is highly expressed in neural stem cells, neurons and astrocytes during early development and the immediate postnatal period (Figure S1). Immunofluorescence staining performed on astrocytes at postnatal day (P0), neurons at embryonic day (E16) and neural stem cells at E13 revealed that EZH2 is indeed highly expressed in astrocytes during the early postnatal phase (Figure 1A,B). Interestingly, our findings, derived from magnetic bead sorting and quantitative PCR (qPCR) analysis of astrocytes isolated from the postnatal brains, indicated that *Ezh2* expression remains stable at a plateau from P0 to P5, after which it begins to decline at P8 (Figure 1C,D). Notably, the P0–P8 period coincides with the peak of astrocyte development, suggesting that EZH2 expression could be critically involved in modulating astrocyte maturation during this critical window of early neurodevelopment.

**FIGURE 2:**
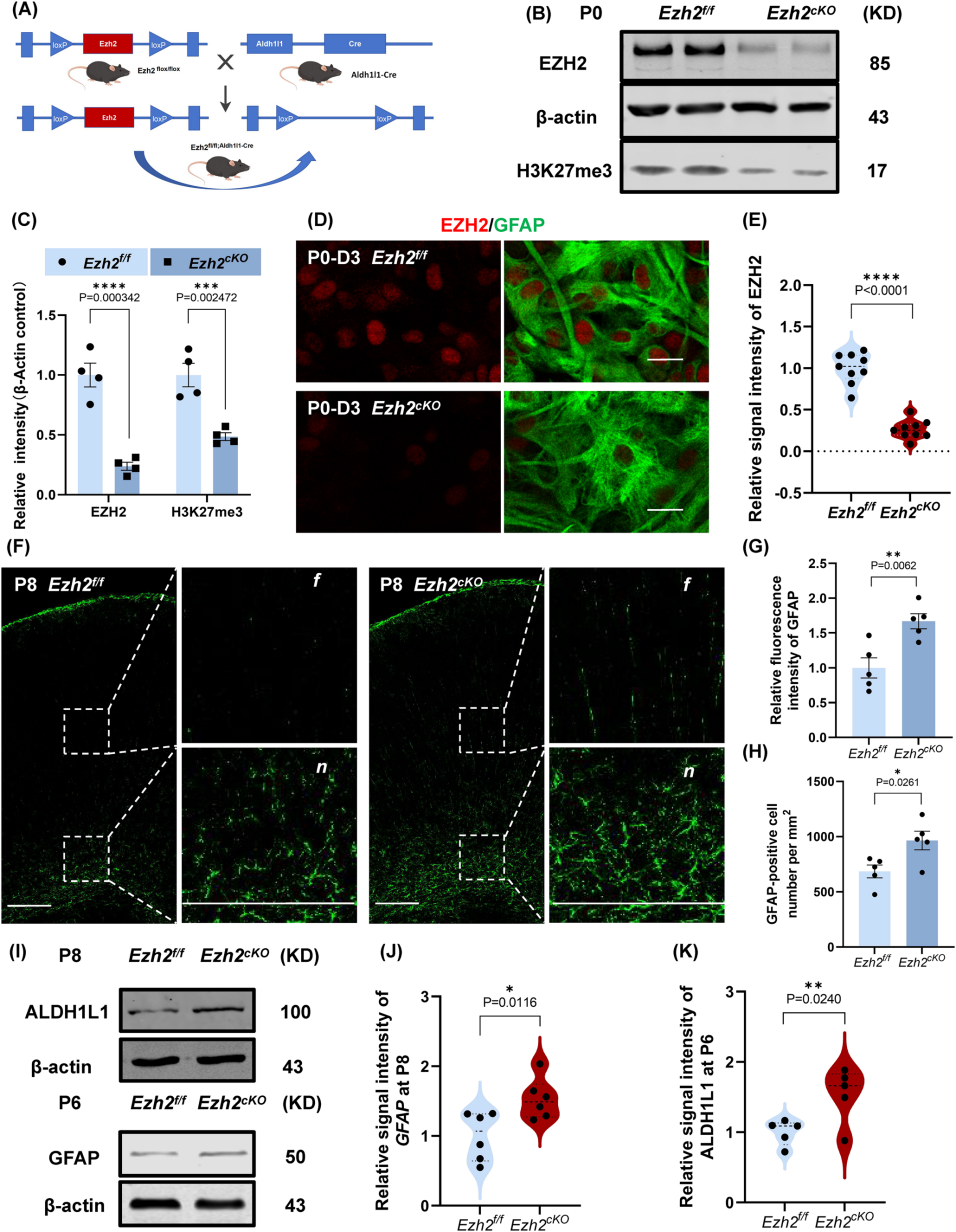
To further explore the role of Ezh2 during the morphogenesis phase of astrocytes, we constructed Ezh2_cKO‐Aldh1l1_ conditional knockout mice. We observed a significant increase in the number of fine processes and the expression of related gliogenesis proteins in astrocytes from Ezh2_cKO‐Aldh1l1_ mice. (A) Schematic diagram of constructing Ezh2 conditional knockout mice in astrocytes. (B) The proteins of EZH2 and H3K27me3 in Ezh2_f/f_ and Ezh2_cKO‐Aldh1l1_ cerebral cortex at P0 were detected and quantified by western blotting. (C) Statistic analysis of the relative intensity of Ezh2 in WB detection, *n* = 4. (D) Immunofluorescence staining of EZH2 in Ezh2_f/f_ and Ezh2_cKO‐Aldh1l1_ cerebral cortex at P0. Scale bars, 20 μm. (E) Quantification showing the decreased fluorescence intensity of EZH2 in Ezh2_cKO‐Aldh1l1_ mice at P0 (*n* = 6 mice per group). (F) Immunofluorescence staining of GFAP in Ezh2_f/f_ and Ezh2_cKO‐Aldh1l1_ cerebral cortex at P8. Scale bars, 200 μm count the fluorescence intensity(f) and cell number(*n*) separately. (G, H) Quantification showing the decreased fluorescence intensity (F‐f) and GFAP‐positive cell number (F‐n) of GFAP in Ezh2_f/f_ and Ezh2_cKO‐Aldh1l1_ mice at P8 (*n* = 5 mice per group). (I) The proteins of GFAP(P8) and ALDH1L1(P6) in Ezh2_f/f_ and Ezh2_cKO‐Aldh1l1_ cerebral cortex were detected and quantified by western blotting. (J) Statistic analysis of the relative intensity of GFAP in WB detection, *n* = 6. (K) Statistic analysis of the relative intensity of ALDH1L1 in WB detection, *n* = 5.

To investigate the specific role of EZH2 in astrocytes, we generated a conditional *Ezh2* knockout (cKO) mouse model by crossing *Ezh2* ^flox/flox^ mice with *Aldh1l1*‐Cre mice, enabling astrocyte‐specific deletion of *Ezh2* (Figure 2A). *Ezh2* deletion was first confirmed by western blot (WB) analysis of astrocytes isolated from the P0 cortex (Figure 2B,C). The results demonstrated a marked reduction in EZH2 expression as well as its catalytic product, H3K27me3, confirming efficient deletion in astrocytes. Further validation was provided by immunofluorescence staining (Figure 2D,E) and qPCR (Figure S2A), both of which corroborated the loss of EZH2 in the knockout mice.

Next, we examined the phenotypic differences between wild‐type and *Ezh2* ^*cKO*^ mice. Cortical area measurements at P2 and P8 showed no significant differences in overall brain morphology (Figure S2B,C). However, when we performed GFAP immunofluorescence staining on cortical tissue at P8, we noted a substantial increase in both the fluorescence intensity and the number of GFAP‐positive astrocytes in the *Ezh2* ^*cKO*^ mice (Figure 2F–H). Protein‐level analysis also revealed increased expression of GFAP and ALDH1L1 in *Ezh2* ^*cKO*^ mice (Figure 2I–K), indicating that EZH2 is a key regulatory factor for astrocyte development and that its deletion leads to an increase in astrocyte number.

### 2.2 Loss of *Ezh2* Affects Astrocyte Morphology in Both Cell Culture and Animal Models

**FIGURE 3:**
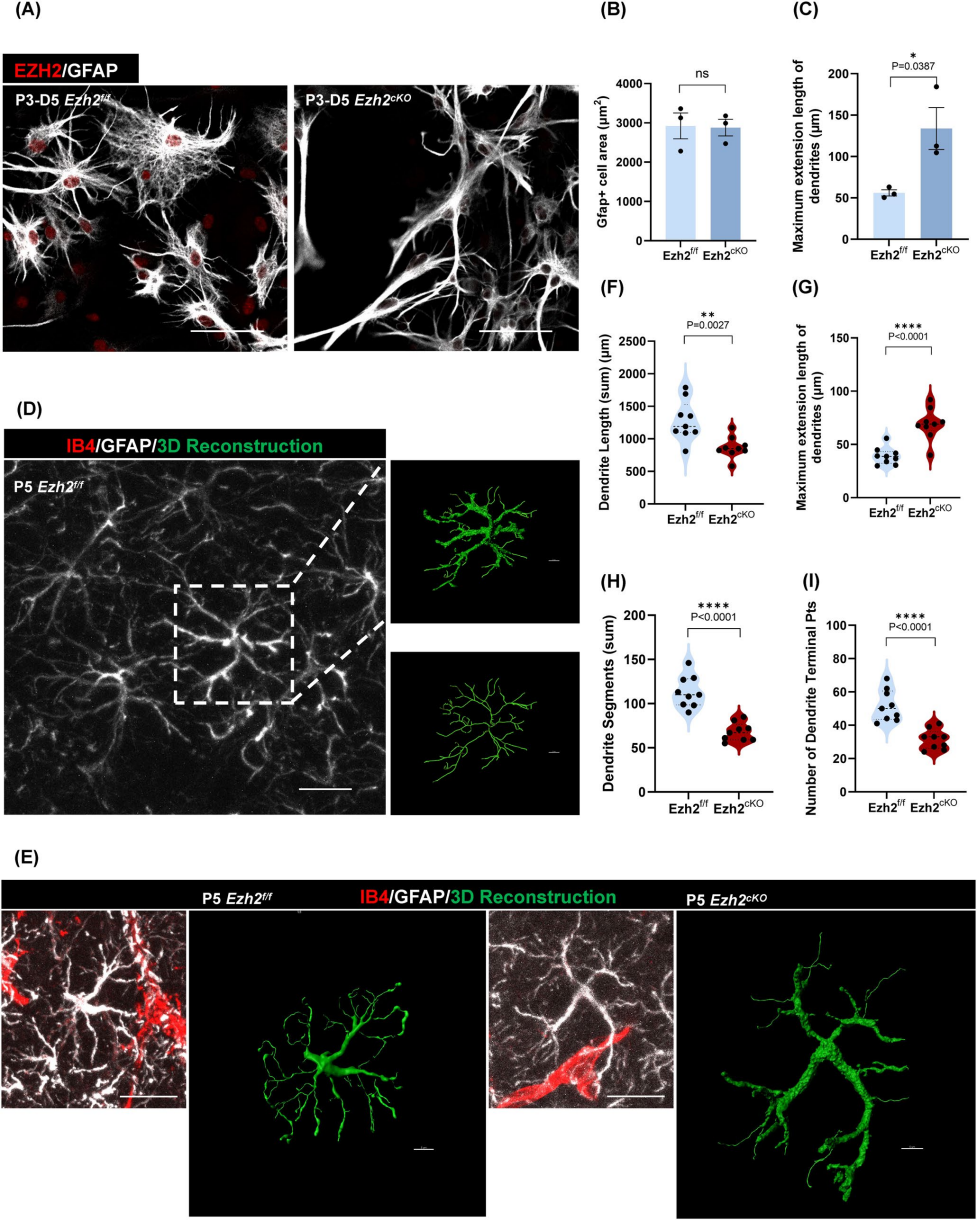
Lack of Ezh2 in astrocytes during early morphological dScrambleelopment affects astrocyte morphology in vitro and in vivo. (A) Immunofluorescent staining for GFAP with primary astrocytes cultured 5 days after isolation from cerebral cortex of mice at P3. Scale bar, 100 μm. (B) Statistic analysis of GFAP+ cell area (μm^2^), *n* = 3. Calculate the average area of all complete cells in each field of view. (C) Statistic analysis of the maximum extension length of dendrites (μm), *n* = 3. (D) Schematic diagram of cell morphology statistics: Immunofluorescence staining and thin‐layer scanning of the cerebral cortex of mice at P5, and then 3D reconstruction and statistics of the GFAP‐positive cell morphology. Scale bar, 20 μm (left), 5 μm (right). (E) Representative morphological images of 3D‐reconstructed cortical astrocytes in Ezh2_f/f_ and Ezh2_cKO‐Aldh1l1_ mice at P5, *n* = 9. Scale bar, 20 μm (left), 5 μm (right). (F) Statistical analysis of the sum of dendritic lengths (μm), *n* = 9. (G) Statistical analysis of maximum extension length of dendrites (μm), *n* = 9. (H) Statistical analysis of dendrite segments, *n* = 9. The schematic diagram of the statistical part can be seen in Figure S3A. (I) Statistical analysis of the number of dendrite terminal points, *n* = 9.

To explore the morphological impact of *Ezh2* deletion on astrocytes, we first isolated primary astrocytes from the P3 cortices of both wild‐type and *Ezh2* ^*cKO*^ mice, cultured them for 3 days, and performed GFAP immunofluorescence staining (Figure 3A). The results demonstrated that astrocytes from *Ezh2* ^*cKO*^ mice exhibited a more elongated morphology compared to wild‐type astrocytes. To quantify these changes, we measured cell area (Figure 3B) and the average length of the longest dendrite (Figure 3C). While no significant differences were observed in cell area, the longest dendrite length in *Ezh2* ^*cKO*^ astrocytes was significantly increased.

To confirm whether these morphological alterations were reflected in vivo, we performed immunofluorescence staining on P8 cortical tissue and utilised Imaris 9.0.1 software for 3D reconstruction and morphological analysis of GFAP‐positive astrocytes (Figures 3D,E and S3A). Our results revealed that, in *Ezh2* ^*cKO*^ mice, astrocytes exhibited a significant reduction in the total dendritic length, branch point number and terminal branch number (Figure 3F,H,I). However, there was no significant difference in the number of primary branches (Figure S3B). In contrast, the maximum dendritic length was significantly increased (Figure 3G), further corroborating our in vitro findings. These results suggest that EZH2 deletion leads to substantial alterations in astrocyte morphology, characterised by a more elongated but less branched dendritic structure.

### 2.3 Decreased Astrocyte Vascular Coverage and Blood–Brain Barrier Dysfunction in *Ezh2* ^*cKO*^ Mice

**FIGURE 4:**
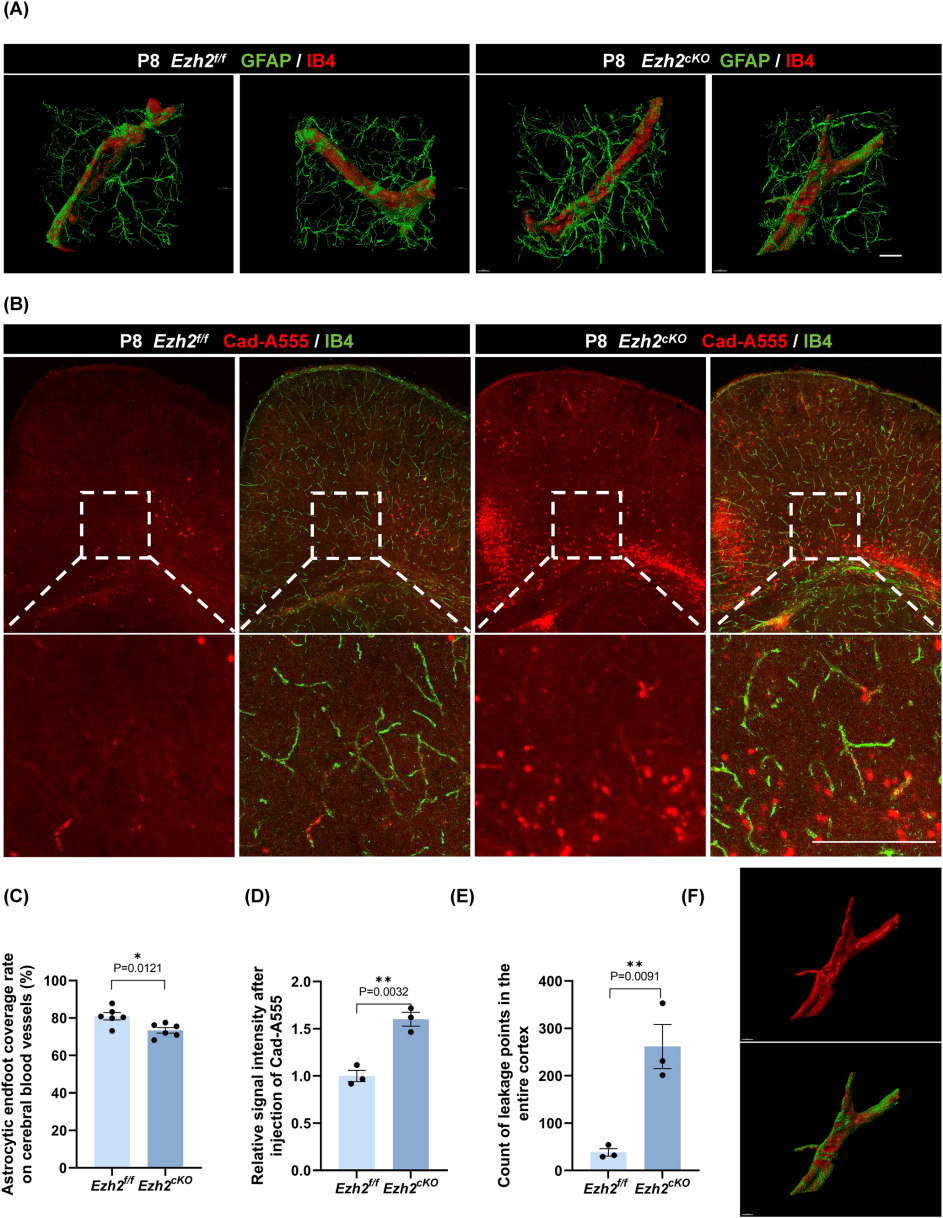
The changes in astrocyte morphology resulted in a decrease in the astrocytic endfoot coverage rate in cKO mice, and further exploration rScrambleealed that the blood–brain barrier of Ezh2_cKO‐Aldh1l1_ group mice was damaged. (A) Representative morphological images of 3D‐reconstructed astrocytic endfeet coverage on cerebral blood vessels in Ezh2_f/f_ and Ezh2_cKO‐Aldh1l1_ mice at P8, *n* = 6. Scale bar, 10 μm. (B) Immunofluorescent staining for Ib4 on the cortex after being injected with Alexa Fluor 555 cadaverine (Cad‐A555) at P8. Scale bar, 200 μm. (C) Statistic analysis of astrocytic endfoot coverage rate on cerebral blood vessels (%), *n* = 6. (D) Statistic analysis of the relative signal intensity after injection with Cad‐A555, *n* = 3. (E) Statistic analysis of the count of leakage points in the entire cortex, *n* = 3. (F) Schematic diagram of the vascular surface area (red) and astrocytic endfeet coverage area (green).

Given the observed changes in astrocyte morphology, we next explored whether these alterations affected the coverage of blood vessels by astrocyte endfeet. Previous studies have shown that changes in astrocyte morphology and function can lead to reduced endfoot coverage and disruption of the BBB in various neurological conditions [26]. To investigate this, we performed immunofluorescence staining on P8 cortical sections of wild‐type and *Ezh2* ^*cKO*^ mice, followed by 3D reconstruction of the astrocyte endfoot and adjacent vasculature (Figure 4A,F). We found that astrocyte endfoot coverage of blood vessels was significantly reduced in *Ezh2* ^*cKO*^ mice (Figure 4C).

We further assessed BBB integrity by injecting Alexa Fluor 555 cadaverine (Cad‐A555) into the peritoneal cavity of P8 mice. After 2 h, we performed IB4 immunofluorescence staining and analysed the distribution of Cad‐A555 in the cortex (Figure 4B). The results revealed that *Ezh2* ^*cKO*^ mice exhibited significantly increased fluorescence intensity of Cad‐A555 and a greater number of leakage points (Figure 4D,E), confirming that BBB permeability was significantly compromised in these mice. This suggests that EZH2 deletion in astrocytes leads to a functional impairment of the BBB, likely due to altered astrocyte morphology and reduced endfoot coverage.

### 2.4 Vascular Abnormalities and Decreased Tight Junction Proteins in *Ezh2* ^*cKO*^ Mice

**FIGURE 5:**
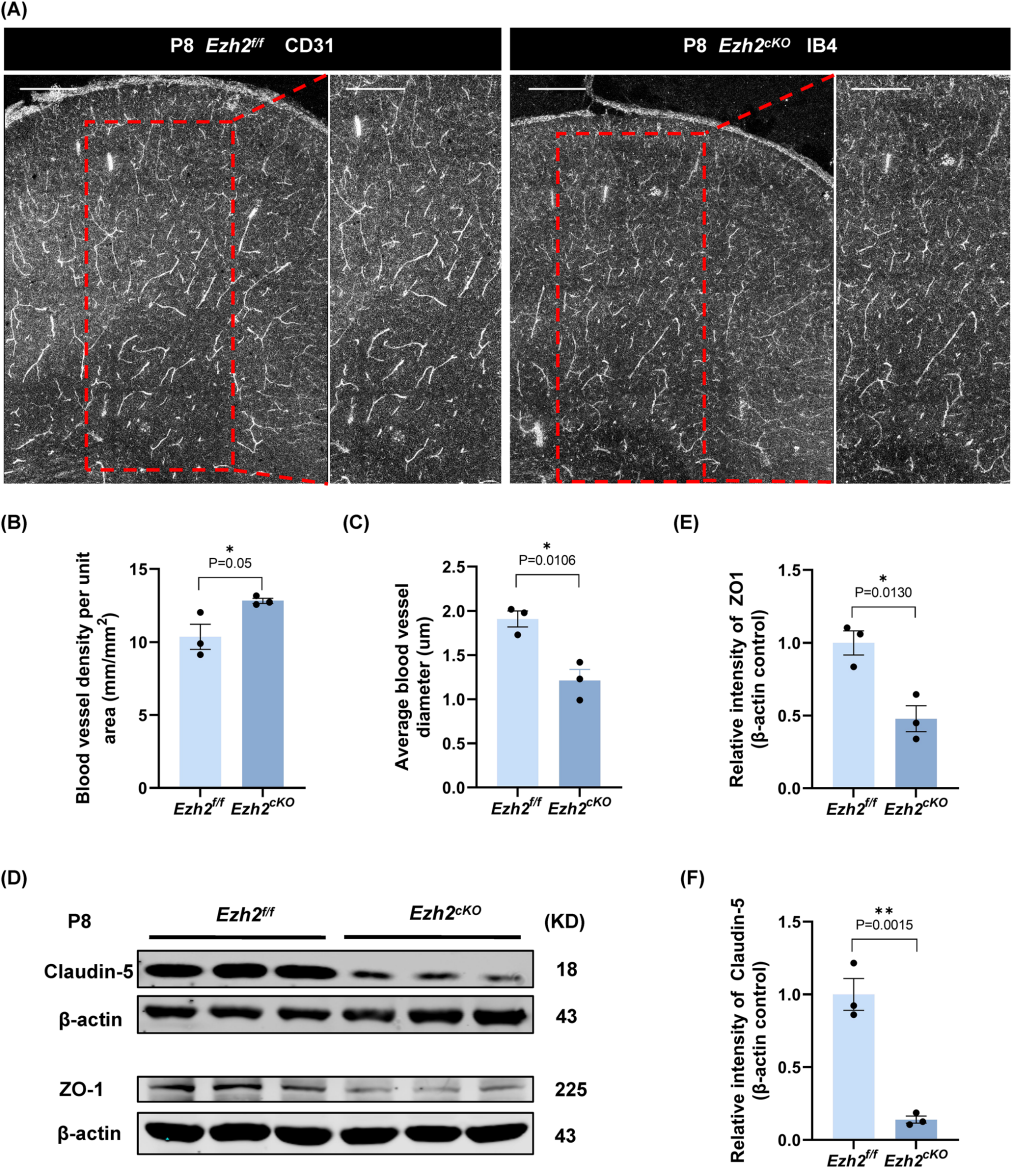
Ezh2_cKO‐Aldh1l1_ mice exhibit abnormal vascular development in the cerebral cortex along with a reduction in tight junction proteins. (A) Immunofluorescent staining for IB4 with mouse brains at P8. Scale bar, 200 μm. (B) Statistic analysis of the blood vessel density per unit area (mm/mm_2_), *n* = 3. (C) Statistic analysis of the average blood vessel diameter (um), *n* = 3. (D) The protein of Claudin‐5 and ZO1 in Ezh2_f/f_ and Ezh2_cKO‐Aldh1l1_ astrocytes of the cerebral cortex was detected and quantified by western blotting, *n* = 3. (E, F) Statistical analysis of the relative intensity of Claudin‐5 and ZO1 in WB detection, *n* = 3.

We further investigated the influence of astrocyte dysfunction on vascular development. For this purpose, we performed IB4 immunofluorescence staining on cortical sections from P8 wild‐type and *Ezh2* ^*cKO*^ mice and quantified vascular density and vessel diameter (Figure 5A). The results revealed vascular abnormalities in the *Ezh2* ^*cKO*^ mice, marked by elevated vascular density but a reduction in vascular diameter (Figure 5B,C). These findings are reminiscent of the compensatory vascular changes observed in clinical conditions such as moyamoya disease, where small vessels proliferate following cerebral ischemia but fail to adequately compensate for the loss of larger vessels [27, 28]. Moreover, we observed a notable decline in the expression of tight junction proteins, Claudin‐5 and ZO1, in the *Ezh2* ^*cKO*^ mice (Figure 5D–F), further suggesting that the disruption of the BBB is a key consequence of *Ezh2* deletion in astrocytes.

### 2.5 Upregulation of DDN and Its Association With H3K27me3 in *Ezh2* ^*cKO*^ Astrocytes

**FIGURE 6:**
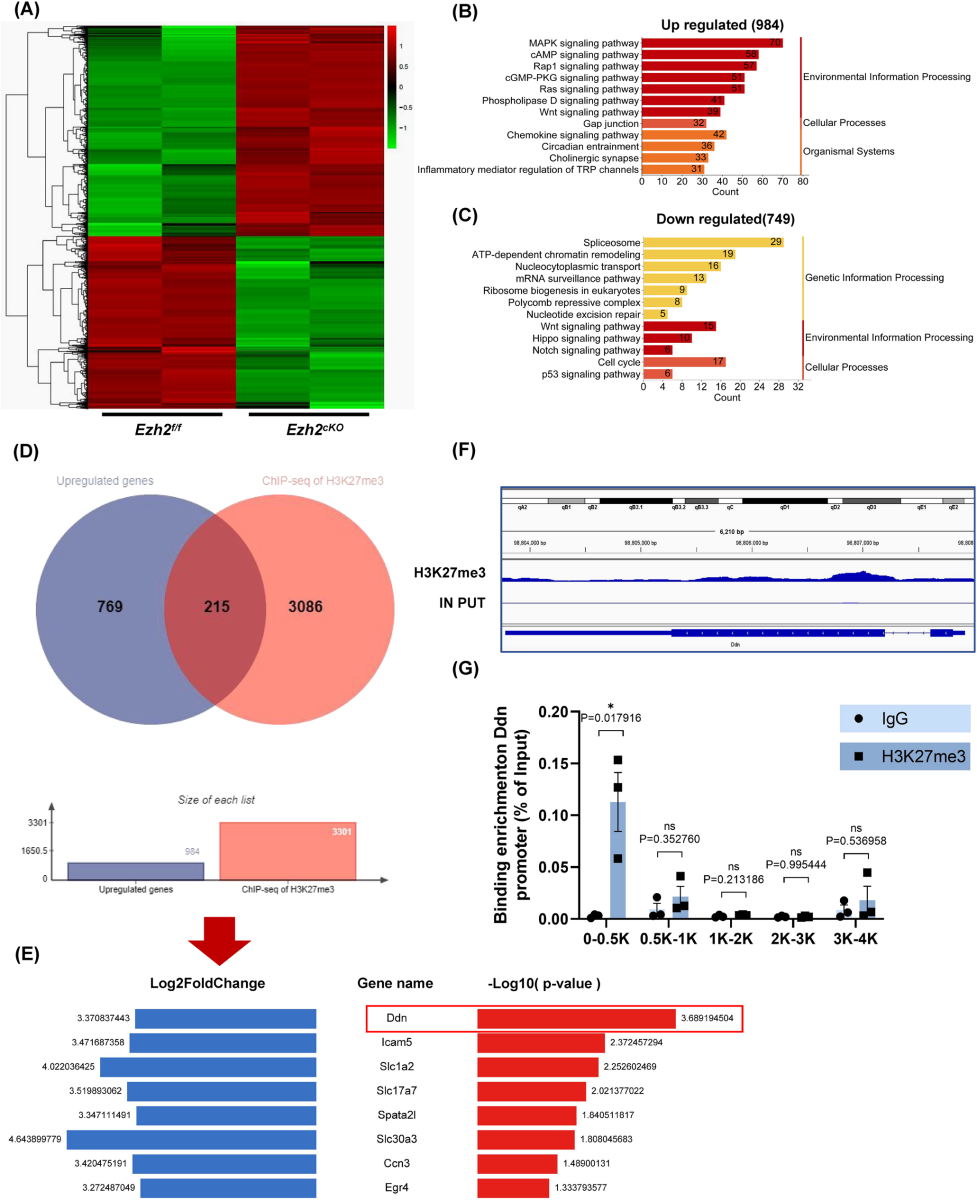
The loss of Ezh2 in astrocytes significantly upregulates Ddn, a downstream target gene of H3K27me3. (A) Heap map of RNA‐sequencing data of astrocytes from P5 mice. (B, C) Upregulated and downregulated signalling pathways by KEGG analysis. (D) By intersecting 984 upregulated genes from RNA‐seq with 3301 peak genes from H3K27me3 ChIP‐seq data, we have filtered out a total of 215 genes. (E) Sort the *p* value and Log_2_ fold change values for 215 intersecting genes. (F) Peaks of Ddn in H3K27me3 ChIP‐seq data. (G) ChIP experiment verified that H3K27me3 binds to the site of the Ddn promoter (*n* = 3 independent experiments).

To explore the molecular mechanisms underlying the effects of *Ezh2* knockout, we performed RNA‐sequencing (RNA‐seq) on astrocytes extracted from the P5 cortex of *Ezh2* ^*f/f*^ and *Ezh2* ^*cKO*^ mice (Figure 6A). Bioinformatics analysis identified 974 genes with significant upregulation and 749 genes with downregulated expression in *Ezh2* ^*cKO*^ astrocytes. Gene ontology and KEGG pathway analysis indicated that the upregulated genes were predominantly involved in the MAPK signalling pathway and biological processes related to the spliceosome (Figure 6B,C). Given that EZH2 is a catalytic subunit of the Polycomb Repressive Complex 2 (PRC2) and a critical epigenetic modulator, its function in gene silencing via the catalysis of H3K27me3 suggests that the upregulation of these genes in the absence of EZH2 reflects the loss of this epigenetic repression [15].

Among the 974 upregulated genes, we identified *Ddn* as the most significantly upregulated gene. To further investigate the relationship between *Ddn* and *Ezh2*, we cross‐referenced these genes with a list of 3301 genes previously shown to interact with H3K27me3, based on ChIP‐seq data for postnatal astrocytes, and identified *Ddn* as a prominent target (Figure 6D–F). Moreover, ChIP assays confirmed that EZH2 directly binds to the promoter region of the *Ddn* gene (Figure 6G), suggesting that *Ddn* is a key transcriptional target of EZH2 in astrocytes.

### 2.6 DDN Regulates Astrocyte Cytoskeleton via the MAPK/ERK Pathway

**FIGURE 7:**
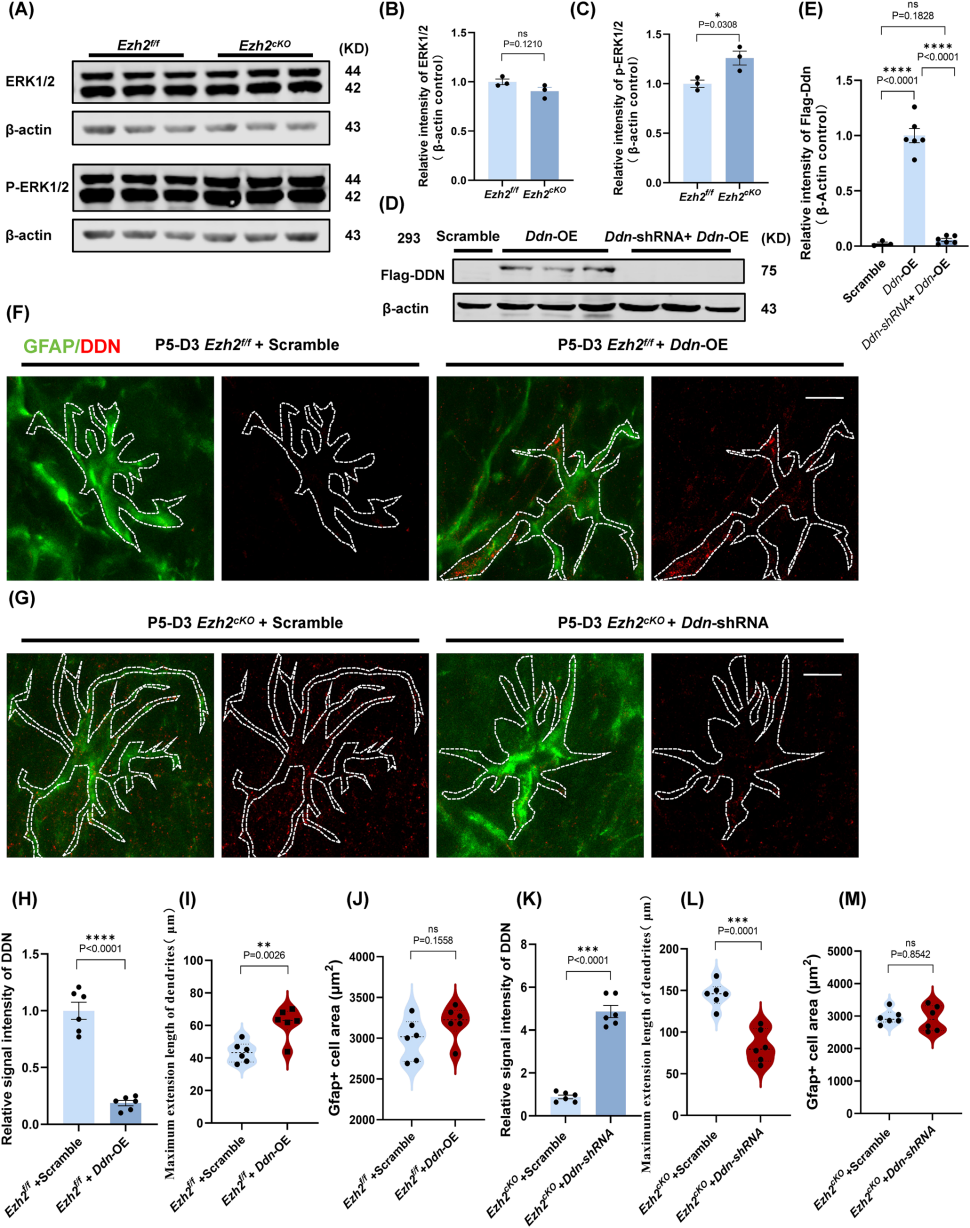
The upregulated Ddn affects the astrocyte cytoskeleton through the MAPK/ERK signalling pathway, thereby altering its cellular morphology. (A) The protein of ERK1/2 and p‐ERK1/2 in astrocytes of Ezh2_f/f_ and Ezh2_cKO‐Aldh1l1_ mice at P5 was detected and quantified by western blotting. (B, C) Statistical analysis of the relative intensity of ERK1/2 and p‐ERK1/2 in WB detection, *n* = 3. (D) The functions of knockdown plasmids (Ddn‐shRNA) and overexpression plasmids (Ddn‐OE) were tested using 293 cell lines, and the knockdown efficiency was verified. The protein of Flag‐Ddn in Scramble and Ddn‐shRNA and (Ddn‐shRNA+Ddn‐OE) was detected and quantified by western blotting. (E) Statistical analysis of the relative intensity of Flag‐DDN in WB detection, *n* = 6 (Ddn‐shRNA and (Ddn‐shRNA+Ddn‐OE)), *n* = 3 (Scramble). (F) Phenotype confirmed by knocking down Ddn gene in astrocytes of Ezh2_f/f_ mice: Immunofluorescent staining for GFAP and Ddn with primary astrocytes cultured for 3 days and infected with Ddn‐shRNA enveloped with virus for 3 days after isolation from the cerebral cortex of Ezh2_f/f_ mice at P5. Scale bar, 50 μm. (G) Overexpression of Ddn in astrocytes of Ezh2_cKO‐Aldh1l1_ mice rescues the phenotype: Immunofluorescent staining for GFAP and Ddn with primary astrocytes cultured for 3 days and infected with Ddn‐shRNA enveloped with virus for 3 days after isolation from the cerebral cortex of Ezh2_cKO‐Aldh1l1_ mice at P5. Scale bar, 50 μm. (H) Statistic analysis of the relative intensity of Ddn in Immunofluorescent staining from (C), *n* = 6. (I) Statistic analysis of GFAP+ cells from (C) area (μm^2^), *n* = 6. Calculate the average area of all complete cells in each field of view. (J) Statistic analysis of the maximum extension length of dendrites (μm) from (C), *n* = 6. (K) Statistic analysis of the relative intensity of Ddn in Immunofluorescent staining from (D), *n* = 6. (L) Statistic analysis of GFAP+ cell from (D) area (μm^2^), *n* = 6. Calculate the average area of all complete cells in each field of view. (M) Statistic analysis of the maximum extension length of dendrites (μm) from (D), *n* = 6.

To further investigate the functional implications of *Ddn* upregulation, we investigated the MAPK/ERK pathway activation in *Ezh2* ^*cKO*^ mice. The results indicated a significant enhancement in the phosphorylation of ERK1/2 (Figure 7A–C). To explore the downstream effects of *Ddn* expression on astrocyte cytoskeletal regulation, we examined the organisation of actin filaments in cultured astrocytes. We found that upregulation of *Ddn* induced notable changes in the astrocytic cytoskeleton, including increased dendritic complexity, which was partially mediated through the MAPK/ERK signalling pathway (Figure 7D,E).

Subsequently, we conducted phenotype rescue studies to further validate the role of *Ddn* in the observed morphological changes. First, we cultured primary astrocytes from wild‐type mice and infected them with lentiviruses carrying *Ddn*‐OE. The results showed that overexpression of *Ddn* in astrocytes recapitulated the morphological phenotypes observed in *Ezh2* ^*cKO*^ astrocytes (Figure 7F,H–J). Next, we cultured primary astrocytes from *Ezh2* ^*cKO*^ mice and infected them with lentiviruses carrying *Ddn–shRNA*. The results demonstrated that the knockdown of *Ddn* in *Ezh2* ^*cKO*^ astrocytes largely restored their morphology to wild‐type levels (Figure 7G,K–M), further supporting the pivotal role of *Ddn* in mediating the morphological changes associated with *Ezh2* deletion.

### 2.7 *Ezh2* ^*cKO*^ Led to Autism‐Like Behavioural in Mice

Given the observed morphological abnormalities and the disruption of BBB integrity, we hypothesised that these changes might contribute to the development of various neurological disorders. For instance, reduced complexity in astrocyte morphology has been associated with disorders such as OCD [13], while compromised BBB integrity and mitochondrial dysfunction are hallmarks of autism spectrum disorders [29]. To assess behavioural changes in *Ezh2* ^*cKO*^ mice, we conducted a series of tests, including the marble burying assay and ultrasonic vocalisation (USV) analysis.

**FIGURE 8:**
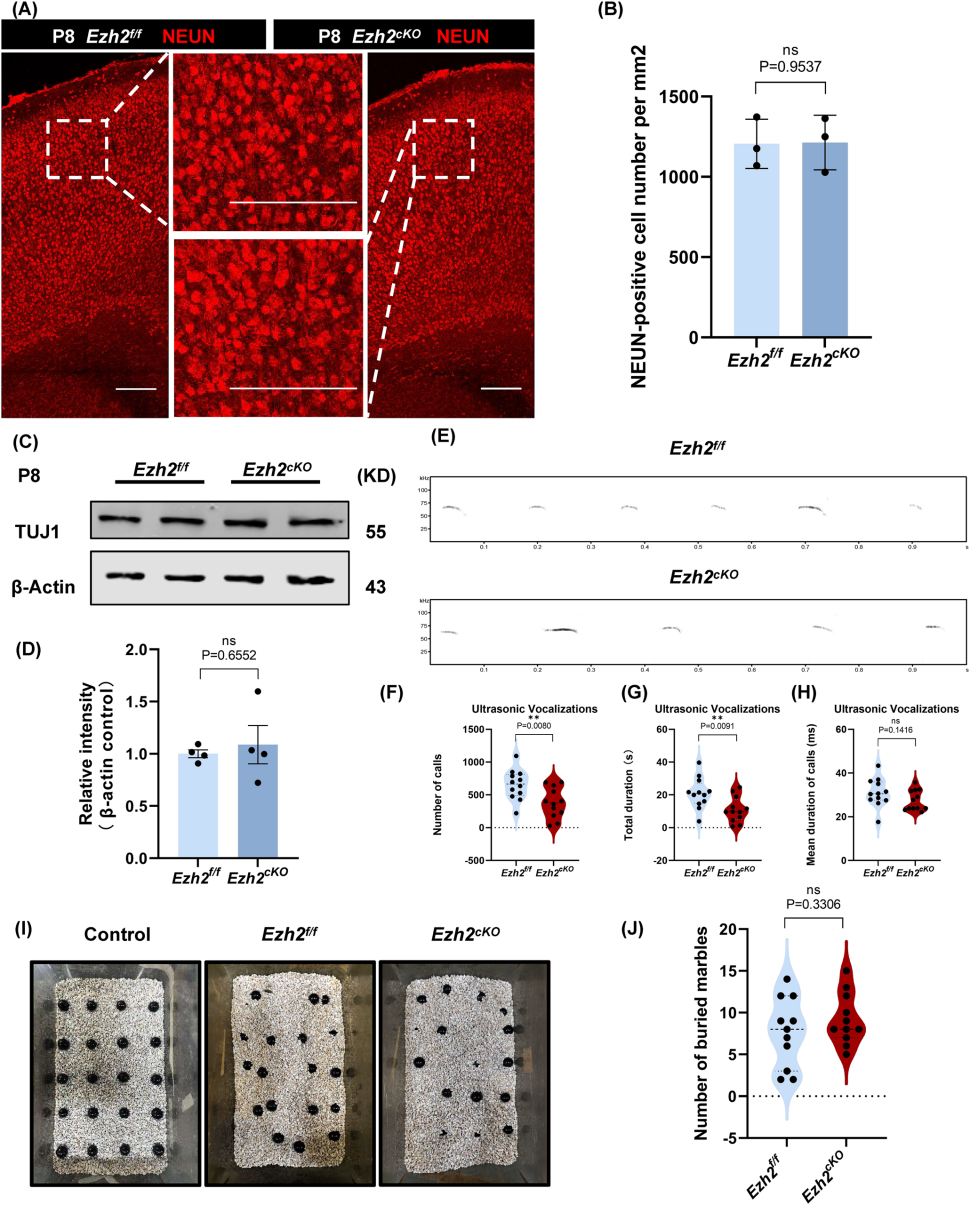
Ezh2_cKO‐Aldh1l1_ showed autistic tendencies, which may be related to the disruption of the blood–brain barrier, but no differences were found in neuronal tests. (A) Immunofluorescent staining for IB4 with mouse brains at P8. Scale bar, 200 μm. (B) Statistic analysis of NEUN‐positive cell number per mm^2^, *n* = 3. (C) The protein of TUJ1 in Ezh2_f/f_ and Ezh2_cKO‐Aldh1l1_ astrocytes of the cerebral cortex was detected and quantified by western blotting, *n* = 3. (D) Statistic analysis of the relative intensity of TUJ1 in WB detection, *n* = 3. (E) Representative spectrogram of the ultrasonic vocalisations (USVs) in isolated pups at P8. (F) Statistical analysis showed that the number of calls (F) and total duration (G) and mean duration of calls (H) (for Ezh2_f/f_ and Ezh2_cKO‐Aldh1l1_ mice, *n* = 12). (I) Representative spectrogram of the marble burying test (MBT) in isolated mice at P30 (Same‐litter same‐sex control). (J) Statistical analysis of the number of buried marbles, *n* = 11.

In the USV experiment, we assessed P8 pups from wild‐type and *Ezh2* ^*cKO*^ mice. *Ezh2* ^*cKO*^ mice exhibited a notable reduction in the total number of vocalisations and the total duration of vocalisation, while the average duration of each vocalisation episode remained unchanged. These findings suggest that *Ezh2* ^*cKO*^ mice may exhibit autism‐like traits (Figure 8E–H). In the marble burying assay, we tested P30 mice and found no significant difference in the number of marbles buried between the wild‐type and *Ezh2* ^*cKO*^ mice, suggesting that the *Ezh2* ^*cKO*^ mice do not exhibit anxiety‐like behaviour (Figure 8I,J). To assess neuronal function in the *Ezh2* ^*cKO*^ mice, we performed NEUN immunofluorescence staining and TUJ1 protein‐level analysis on cortical sections from P8 mice (Figure 8A,C). Both the staining and protein‐level measurements exhibited no significant variation across the groups (Figure 8B,D), suggesting that neuronal development is not markedly affected by the deletion of *Ezh2* in astrocytes.

## 3 Discussion

The complexity of astrocyte morphology is very important for maintaining the normal physiological functions of the nervous system. Understanding the morphogenesis of astrocytes is the basis for understanding the functions of their morphological complexity. The first week after birth is the early stage of astrocyte morphological development in mice, which is mainly responsible for branch extension and establishment of endfeet. It is also the period when morphological changes are most significant [30, 31]. Therefore, this paper mainly focuses on the early morphogenesis of astrocytes. The second and third weeks after birth are the middle and final stages of morphological development, respectively, which are mainly to improve morphological details, coordinate cell distribution, functional development, etc. [32]. Our preliminary experiments revealed that the stable expression plateau of EZH2 in astrocytes closely coincides with the early stages of astrocytic morphogenesis.

The results of this study also revealed the important function of EZH2 in the early morphological development of astrocytes and, for the first time, associated its loss with morphological changes in the development of the nervous system, damage to the BBB function and autism‐like behaviours. By constructing the *Ezh2* ^*cKO*^ mouse model, we found that knocking out *Ezh2* in astrocytes significantly increased the number of astrocytes and the expression level of GFAP, suggesting that *Ezh2* may play a role in inhibiting excessive extension of dendrites in the early stage of astrocyte morphogenesis. In addition, the morphological changes caused by the lack of *Ezh2* further affected the number and structural integrity of astrocyte endfeet, thereby exhibiting BBB leakage. It is known that the coverage of astrocyte endfeet helps maintain the integrity of the blood–brain barrier [26]. The reduced coverage of astrocytic endfeet on blood vessels and the disruption of the BBB observed in *Ezh2* ^*cKO*^ mice indicate that *Ezh2* plays an indirect but critical role in maintaining the integrity of the BBB. Subsequently, we also detected decreased levels of tight junction proteins in endothelial cells of *Ezh2* ^*cKO*^ mice, which also supported the disruption of BBB function. The reason for this result may be, on the one hand, the destruction of the BBB structure after the astrocytic endfoot coverage rate decreases, and, on the other hand, it may also be due to a decrease in the secretion of neurotrophic factors. Both possibilities can explain the phenomenon of small blood vessels and compensatory hyperplasia in Ezh2^cKO^ mice. However, no difference in the expression of neurotrophic factor‐related transcription factors was found in our RNA‐seq results. Therefore, the exact mechanism of blood–brain barrier disruption needs further exploration. This study found that the morphological changes of astrocytes after the lack of Ezh2 were similar to those of reactive astrocytes, and we observed that the increased expression of GFAP is also one of the markers of reactive astrocytes, so we also suspected that the phenotype of cell morphological changes was actually the result of astrocytes differentiating into reactive astrocytes. However, we did not find statistical differences in the expression of related markers (S100a10, C3, Plp1, Serping1, etc.) in the RNA‐seq sequencing results, so this conjecture is not valid.

**FIGURE 9:**
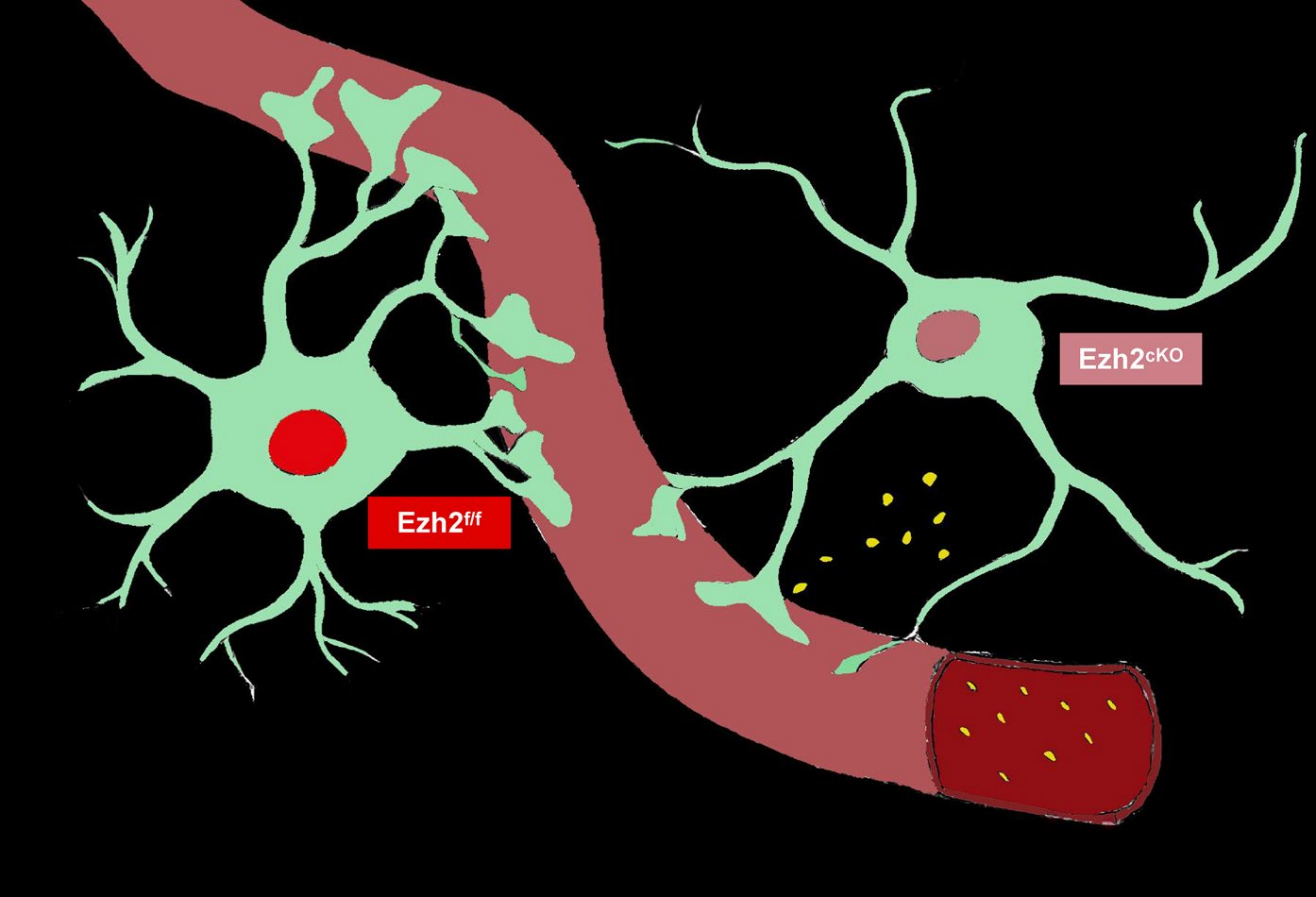
Mechanism diagram. Ezh2‐deficient astrocytes showed morphologically more slender but sparse dendrites, resulting in a reduced endfoot coverage and also causing disruption of the blood–brain barrier.

Furthermore, we observed that the *Ddn* gene, regulated by H3K27me3, was significantly upregulated following *Ezh2* depletion, influencing the astrocyte cytoskeleton through the MAPK/ERK signalling pathway and subsequently altering cell morphology. In the very limited reports on *Ddn*, we know that DDN is a postsynaptic protein that was first discovered in mouse neurons and is mainly found in the forebrain and hippocampus. Dendritic proteins exist in the cell bodies and dendrites of neurons and are important for maintaining the skeletal structure of cells [33, 34, 35]. Although DDN has been discovered in relatively few locations, it is associated with the cytoskeleton in the tissues where it has been discovered [33, 34, 35, 36]. In 2006, Kawata et al. [35] found that dendritic proteins are also located in the kidneys. In their study, they found that dendritic proteins are connected to the cytoskeletal proteins S–S–SCAM and CIN85, which are important for maintaining cell morphology. By querying the public Tabula Muris database (Figure S4), it was found that *Ddn* was mainly expressed in neurons and that the expression level in astrocytes was very low. Therefore, our experimental results can be interpreted as follows: the loss of EZH2 in astrocytes leads to an abnormal increase in *Ddn* expression, which subsequently affects cytoskeletal dynamics and results in aberrant morphological extension. This ultimately affects the coverage of the astrocytic endfeet on the blood vessels, leading to damage to the BBB, as shown in the mechanism diagram (Figure 9).

Finally, we observed that *Ezh2* ^*cKO*^ mice exhibited autism‐like behaviours, although we did not detect abnormalities in neuronal development. However, the abnormal astrocytic morphology and BBB disruption observed in *Ezh2* ^*cKO*^ mice could also reasonably contribute to this pathological condition, and an increasing number of similar reports have confirmed our view [29, 37, 38, 39].

## 4 Future Research Directions

### 4.1 Mechanistic Insights

Further investigation is needed to elucidate how DDN regulates the astrocytic cytoskeleton and morphology by modulating the MAPK/ERK signalling pathway.

### 4.2 Astrocytes and Autism

Additional studies should explore the role of astrocytes in the pathogenesis of autism, with a particular focus on their interactions with neurons and the potential impact on neural network development and function.

### 4.3 BBB Repair Strategies

Future research should examine whether restoring Ezh2 function or targeting the MAPK/ERK pathway could effectively reverse BBB damage and autism‐like behaviours observed in the Ezh2cKO mouse model.

## 5 Materials and Methods

### 5.1 Animals

The experimental animals used in this study included adult and pregnant mice of the C57BL/6J and ICR (CD1) strains, purchased from Charles River Laboratory Animal Technology Co. Ltd. (Beijing) and SPF Biotechnology Co. Ltd. (Beijing). The Ezh2^flox/flox^ (*Ezh2* ^*f/f*^) conditional knockout mice were obtained from The Jackson Laboratory (USA) (Strain #: 022616). Aldh1l1‐icre mice were obtained from GemPharmatech Co. Ltd. (Jiangsu, China) (Strain ID: T006926). Ezh2^flox/flox^ (*Ezh2* ^*f/f*^) mice were bred with *Aldh1l1‐icre* mice to produce *Ezh2* ^*cKO‐Aldh1l1*^ (*Ezh2* ^*cKO*^) mice. All mice used in this study were housed under specific pathogen‐free (SPF) conditions in the animal facility of the Innovation Institute of Stem Cell and Regenerative Medicine, Chinese Academy of Sciences. All experimental procedures involving mice were approved by the Institutional Animal Care and Use Committee (IACUC) of the Institute of Zoology, Chinese Academy of Sciences (Approval No. IOZ20190080). The PCR primers used for genotyping are listed in Table 1.

**TABLE 1:** All primer sequences used in this study (gene identification‐related primers were provided by the knockout mouse company; qPCR primers were designed by the NCBI webpage and provided by harvard primer bank; ChIP primers were designed by SnapGene Viewer 5.2.4 software).

Genotype identification
ALDH1L1‐cre
Forward primer: CCTCAGGGCTTGGTTTCTCATC
Reverse primer: CTTCCAGGTGTGTTCAGAGAAGG
Ezh2
Forward primer: CCTCAGGGCTTGGTTTCTCATC
Reverse primer: CTTCCAGGTGTGTTCAGAGAAGG
qPCR
ALDH1L1
Forward primer: CAGGAGGTTTACTGCCAGCTA
Reverse primer: CACGTTGAGTTCTGCACCCA
GFAP
Forward primer: CGGAGACGCATCACCTCTG
Reverse primer: AGGGAGTGGAGGAGTCATTCG
DDN
Forward primer: GACCCTGGGGACTAAGCGA
Reverse primer: ACATCCCGGTAGATTCGAGGA
EZH2
Forward primer: AGTGACTTGGATTTTCCAGCAC
Reverse primer: AATTCTGTTGTAAGGGCGACC
β‐actin
Forward primer: AGTGTGACGTTGACATCCGT
Reverse primer: AGCTCAGTAACAGTCCGCCTA
ZO1
Forward primer: GCTTTAGCGAACAGAAGGAGC
Reverse primer: TTCATTTTTCCGAGACTTCACCA
Claudin 5
Forward primer: TAAGGCACGGGTAGCACTCA
Reverse primer: GGACAACGATGTTGGCGAAC
CD31
Forward primer: GGAAGTGTCCTCCCTTGAGC
Reverse primer: GGAGCCTTCCGTTCTTAGGG
ChIP
DDN
0–0.5 K
Forward primer: TGTTCTCACCACTCCCTGTCG
Reverse primer: CCCTTTGCTTTCCTGCTTGT
0.5–1 K
Forward primer: ACGCCCGGCTTGATTGTT
Reverse primer: ACCCTGGATGAGCACATGACTA
1–2 K
Forward primer: GTAGACCAGGCTGGCCTTCA
Reverse primer: CTGGCCTCCTCAGGCAACTA
2–3 K
Forward primer: ATTTCCCTAACATGTCAGGT
Reverse primer: GAAAGGCAAGGGAATTGTGG
3–4 K
Forward primer: AAAGTCAGGAGCAGCAAATGG
Reverse primer: TGGTAATGTTAGTGGCAGCACA

### 5.2 Plasmid Constructs

Total RNA was isolated from embryonic mouse brains and subsequently reverse‐transcribed using the Fast Quant RT Kit (TIANGEN). The mouse *Ddn* gene was amplified via PCR and inserted into the pCDH‐copGFP vector, which was engineered to include a 3 × Flag tag. The sequence for mouse *Ddn*‐shRNA was retrieved from the Sigma website and subcloned into the pSicoR‐mCherry vector.

### 5.3 Isolation and Purification of Astrocytes

The astrocyte isolation process was strictly performed according to the instructions provided in the Anti‐GLAST (ACSA‐1) MicroBead Kit manual (please refer to www.miltenyibiotec.com for all data sheets and protocols).

### 5.4 Immunofluorescence Staining (IF)

Tissue samples were fixed in 4% paraformaldehyde (PFA) for 24 h, followed by dehydration in 30% sucrose for an additional 24 h. After sectioning, the brain slices were further fixed in PFA (4%) for 30 min and then rinsed two to three times with PBS containing 0.1% Triton X‐100. To prevent nonspecific binding, the sections were blocked with 5% bovine serum albumin (BSA) for 1 h. Subsequently, the slices were incubated with the primary antibody overnight at 4°C. After three additional washes, secondary antibody incubation was performed for 1 h at room temperature. IF images were captured using a Zeiss LSM 880 confocal microscope.

### 5.5 Quantitative Real‐Time PCR Analysis

Total RNA was isolated from mouse cerebral cortex or cultured cells using TRIzol reagent (Invitrogen, 15596018) according to the manufacturer's protocol. Complementary DNA (cDNA) was synthesised with the FastQuant RT Kit (TIANGEN, KR106‐02). Quantitative real‐time PCR (qRT‐PCR) analysis was conducted using the SuperReal PreMix Plus Kit (SYBR Green I) (TIANGEN, FP205‐02) on an ABI 7500 Real‐Time PCR System (Applied Biosystems). Expression levels of β‐actin or GAPDH were used as internal references for data normalisation. All qRT‐PCR experiments were performed in a minimum of three independent biological replicates. The related primer sequences used for real‐time PCR are listed in Table 1.

### 5.6 Western Blotting

Mouse brain tissues or cultured cells were initially lysed by sonication in RIPA buffer (Solarbio) containing a protease inhibitor cocktail and PMSF. Following centrifugation, protein concentrations were quantified using the Pierce BCA Protein Assay Kit. The extracted proteins were subsequently separated on 10% or 12% SDS‐PAGE gels and transferred onto nitrocellulose (NC) membranes. To block nonspecific binding, membranes were incubated with 5% skim milk or BSA prepared in PBST (1 M PBS with 0.05% Tween‐20) for 1 h at room temperature. Primary antibody incubation was performed overnight at 4°C on a shaker. The next day, membranes were exposed to secondary antibodies for 120 min at room temperature. Protein bands were then detected and analysed using the Odyssey imaging system (LI‐COR Biosciences).

### 5.7 Chromatin Immunoprecipitation (ChIP)

ChIP was conducted using the H3K27me3 antibody (C36B11, Rabbit mAb #9733, CST) to evaluate H3K27me3 enrichment at the promoter region of the *Ddn* gene. Purified mouse cortical astrocytes were crosslinked with 1% formaldehyde for 15 min at room temperature, followed by quenching with 2.5 M glycine for 10 min to halt the crosslinking reaction. The cells were lysed in RIPA buffer supplemented with protease inhibitors, and chromatin was fragmented via sonication to obtain sheared DNA. The chromatin lysate was subsequently incubated overnight at 4°C with gentle rotation. On the following day, magnetic beads pre‐bound with the H3K27me3 antibody for 4 h were added to the chromatin lysate and incubated overnight at 4°C with continuous rotation. To eliminate nonspecific interactions, the beads underwent three sequential washes using low‐salt and high‐salt wash buffers. Chromatin complexes were eluted, and crosslink reversal was performed by heating the samples at 65°C overnight. Genomic DNA was subsequently purified using the TIANamp Genomic DNA Kit (TIANGEN, DP304‐03) and analysed by quantitative real‐time PCR (qPCR) using primers targeting the *Ddn* promoter region on an ABI 7500 Real‐Time PCR System. The relative enrichment of H3K27me3 was determined by normalising to input DNA. All ChIP assays were performed using at least three independent biological replicates. The primer sequences utilised for qPCR are provided in Table 1.

### 5.8 Ultrasonic Vocalisation (USVs)

USV assessments were conducted between 13:00 and 16:00. On postnatal day 8 (P8), Ezh2^f/f^ and *Ezh2* ^*cKO*^ pups were individually separated from their mothers and littermates and then placed in a soundproof chamber for a duration of 5 min. USVs were captured and visualised using the Avisoft Recorder software along with an Ultra Sound Gate Condenser Microphone CM16 (Avisoft Bioacoustics, Berlin). The microphone, calibrated for a frequency range of 25–140 kHz (±6 dB), was positioned 10 cm above the pups. Spectrogram settings included a frequency resolution of 488 Hz and a time resolution of 0.512 ms. Vocalisation‐related parameters were recorded and analysed during the testing period.

### 5.9 Marble Burying Test

The marble burying test was conducted using a standard mouse cage and 20 black glass marbles, each with a diameter of 15 mm. Prior to use, the marbles were thoroughly cleaned and dried. Before each trial, the cage was filled with a 5‐cm layer of fresh bedding, and the 20 marbles were systematically arranged in four rows. The test mouse was gently placed in one corner of the cage and allowed to explore freely for 30 min. After the trial, photographs were taken, and the number of marbles buried with at least two‐thirds of their surface covered was recorded.

### 5.10 RNA‐Sequencing and Data Analysis

Cortical astrocytes from *Ezh2* ^*f/f*^ and *Ezh2* ^*cKO*^ mice at P5 were isolated using magnetic bead sorting, following a previously described protocol. Total RNA was extracted from the purified astrocytes using the TIANGEN RNAprep Pure Micro Kit (#DP420). After quality assessment and cDNA library preparation, high‐throughput sequencing was conducted using the Illumina HiSeq 2500 platform. The raw sequencing data generated in this study have been deposited in the NCBI GEO database under the accession number GSE283622.

### 5.11 Three‐Dimensional (3D) Reconstruction

Confocal images were captured from 40‐μm brain sections stained with IB4 and GFAP using a 40× oil immersion objective lens, with a *z*‐step size of 0.85 μm. GFAP‐positive cells and blood vessels were tracked from the acquired 2D confocal images using Imaris software (version 9.0.1). For each image, 10 cells were randomly chosen for analysis. The three‐dimensional (3D) reconstruction of astrocytes and blood vessels was performed using the ‘Surface’ and ‘Filaments’ tools in Imaris 9.0.1 software.

### 5.12 H3K27me3 ChIP‐Seq Data Processing and Analysis

The raw ChIP‐seq data were first processed using fastp for quality control and adapter trimming to remove low‐quality reads and adapter sequences, ensuring data quality. The trimmed reads were aligned to the reference genome using BWA‐MEM, and the resulting alignment files were processed with samtools for format conversion, sorting, and indexing to generate standardised BAM files for downstream analysis. BAM files from the experimental and control groups were input into MACS2 for peak calling, identifying significantly enriched regions associated with the target modification, along with statistical results and signal intensity distribution files. Subsequently, the peak calling results were further analysed with MACS2 to calculate signal enrichment, generating visualisation files compatible with genome browsers. Additionally, the called peaks were annotated using bedtools in combination with genome annotation files to identify regions associated with genomic features.

### 5.13 Quantification and Statistical Analysis

Statistical analyses were conducted using GraphPad Prism 9.0 software, with results presented as mean ± SEM. For comparisons between two groups, unpaired two‐tailed Student's *t*‐tests were applied, while one‐way ANOVA followed by Tukey's post hoc test was utilised for multiple group comparisons. Statistical significance was defined by the following *p* value thresholds: *p* < 0.05, *p* < 0.01, *p < 0.001 and **p < 0.0001. Non‐significant differences were indicated as n.s.

### 5.14 Antibodies

The following primary antibodies and dilutions were used for immunostaining and WB: EZH2 (CST, 5246, Rabbit, 1:1000); H3K27me3 (Cell Signalling, C36B11, 1:50 (ChIP)， 1:1000 (WB)); *Ddn* (Sigma, AB15299‐I, Rabbit, 1:250); Aldh1l1 (Abcam, ab56777, Mouse, 1:1000); GFAP (Dako, Z0334, Rabbit, 1:1000); IB4 (Vector Labs, B‐1205, Strep, 1:500); ZO‐1 (Invitrogen, 40‐2200, Rabbit, 1:1000); Claudin5 (Invitrogen, 352500, Mouse, 1:1000); NeuN (Abcam, ab177487, Rabbit, 1:1000); Tuj1 (Millipore, MAB1637, Rabbit, 1:1000); Nestin Millipore, (MAB353, Mouse, 1:1000); MAP2 (Millipore MAB378, Mouse, 1:500); IgG (Bioss, bs‐0295, Rabbit, 1:1000); Flag (Sigma, F7425, Mouse, 1:2000); β‐Actin (Proteintech, 20536‐1‐AP, Rabbit, 1:10000); β‐Actin (Proteintech; 60008‐1‐Ig Mouse, 1:2000); IgG (Bioss, bs‐0295p; Rabbit, 1:1000). Secondary antibodies: for immunostaining: DAPI (2 mg/mL; Sigma; D9542); Alexa Fluor 488, Cy3, or Cy5 (Jackson ImmunoResearch, 1:1000). Isolation of astrocytes using the ACSA‐1 MicroBead Kit (Miltenyi Biotec, 130‐095‐826).

## Author Contributions

Xinghua Zhao, Mengtian Zhang, Fen Ji and Libo Su conceptualised the experiment. Mengtian Zhang, Yanyan Wang, Chenxiao Li and Chenqi Yuan provided assistance with the experimental procedures. Shukui Zhang and Wenzheng Zou assisted with data processing. Wenzheng Zou and Yuqing Lv provided writing suggestions. Yufei Gao, Jianwei Jiao, Jinnan Wang, Fen Ji and Nan Wang supervised the project and secured funding.

## Conflicts of Interest

The authors declare no conflicts of interest.

## Supporting information


Data S1.

## Data Availability

Research data are not shared.
