# Supplementary material for: Ezh2 Regulates Early Astrocyte Morphogenesis and Influences the Coverage of Astrocytic Endfeet on the Vasculature
Source: Cell Prolif. 2025 Feb 28;58(8):e70015. doi: 10.1111/cpr.70015 (PMC12336451; doi:10.1111/cpr.70015)
Supplement: Supplementary file 1 — Data S1. [file CPR-58-e70015-s001.pdf]

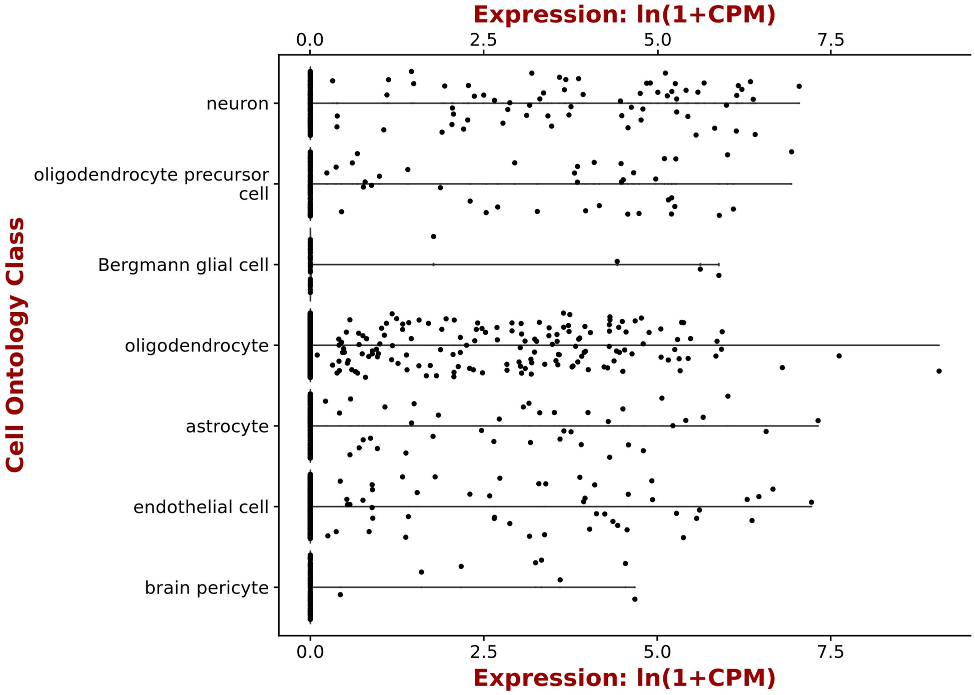

Tissue	Mean	Median	Median > 0	# cells	% 0	% > 0
neuron	0.92	0	3.76	281	76.16	23.84
oligodendrocyte precursor cell	0.67	0	3.97	203	80.79	19.21
Bergmann glial cell	0.44	0	5.02	40	90.00	10.00
oligodendrocyte	0.28	0	3.03	1574	90.09	9.91
astrocyte	0.28	0	3.17	432	90.97	9.03
endothelial cell	0.22	0	3.29	715	93.15	6.85
brain pericyte	0.15	0	3.29	156	94.87	5.13

Figure. S1 Expression of *Ezh2* in various cells of Brain Non-Myeloid. Data Source：https://tabula-muris.sf.czbiohub.org/visualizations.

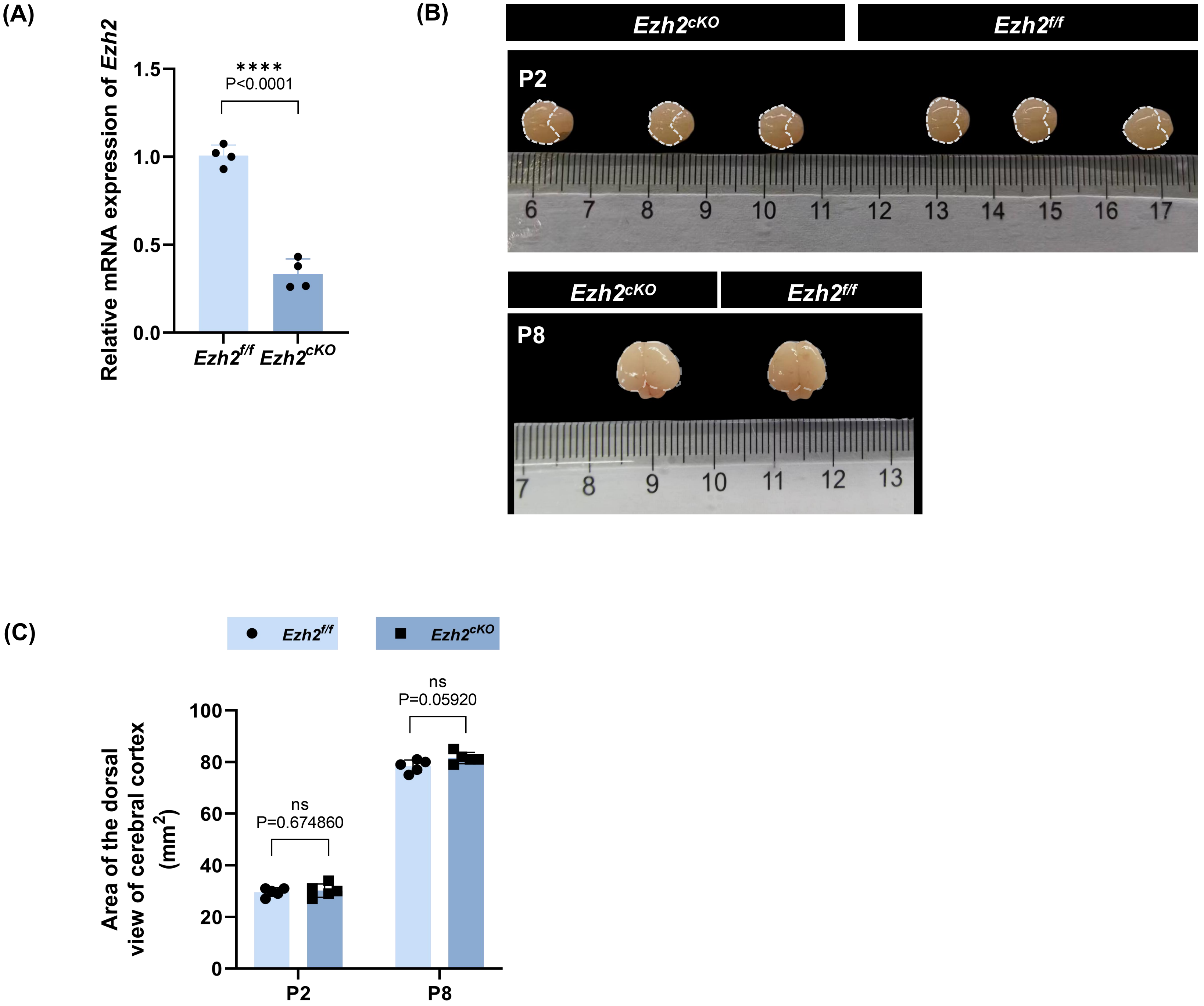

Figure.s2

(A) Verify knockout efficiency: Statistic analysis of relative intensity of EZH2 in q-PCR detection, n = 4.
(B) Macroscopic image of *Ezh2*$^{f/f}$ and *Ezh2*$^{cKO\text{-}Aldh1l1}$ cerebral cortex at P2 and P8.
(C) Statistic analysis of area of *Ezh2*$^{f/f}$ and *Ezh2*$^{cKO\text{-}Aldh1l1}$ cerebral cortex at P2 and P8 from top view, n = 5.

(A)

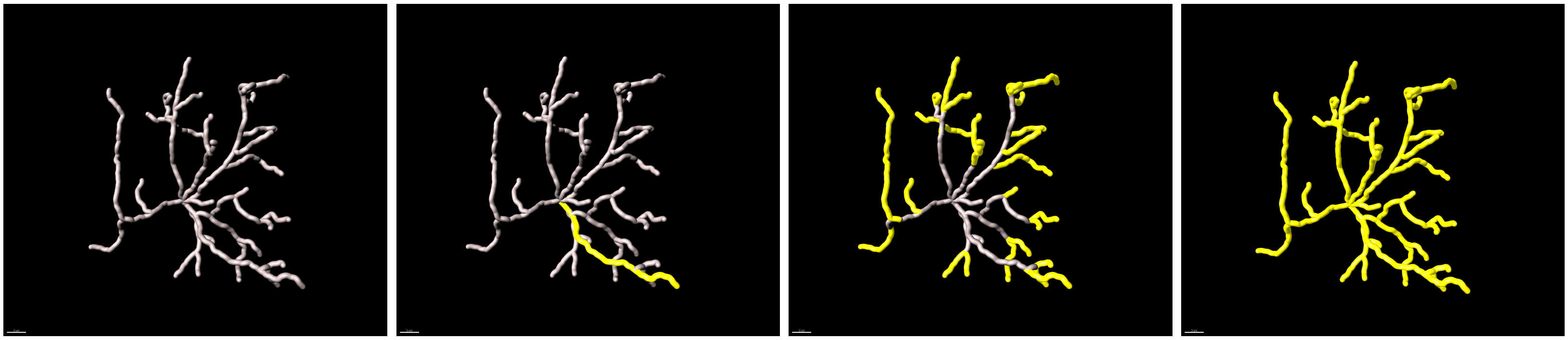

(B)

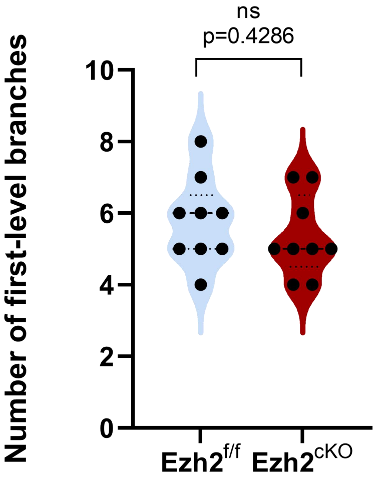

Figure. S3

(A) Schematic diagram of dendritic morphology statistics: from left to right, the yellow parts represent Maximum extension length of dendrites, Number of Dendrite Terminal Pts, and Dendrite Length (sum)
(B) Statistical analysis of Number of first-level branches, n = 9.

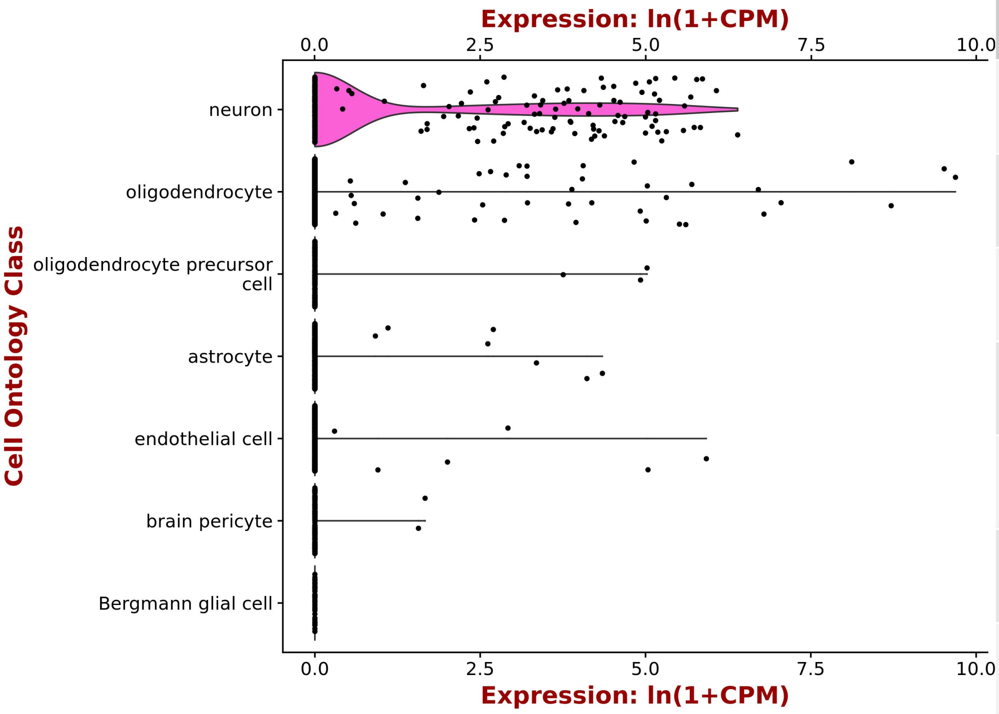

Tissue	Mean	Median	Median > 0	# cells	% 0	% > 0
neuron	1.23	0	3.87	281	67.26	32.74
oligodendrocyte	0.10	0	3.84	1574	97.40	2.60
oligodendrocyte precursor cell	0.07	0	4.92	203	98.52	1.48
astrocyte	0.04	0	2.70	432	98.38	1.62
endothelial cell	0.02	0	2.46	715	99.16	0.84
brain pericyte	0.02	0	1.62	156	98.72	1.28
Bergmann glial cell	0.00	0	NA	40	100.00	0.00

Figure. S4

Expression of *Ddn* in various cells of Brain Non-Myeloid. Data Source：https://tabula-muris.sf.czbiohub.org/visualizations.
